# Hypertension and hyperkalemia associated with Pro701Leu mutation in the NR3C2 gene: A case report

**DOI:** 10.1097/MD.0000000000041978

**Published:** 2025-05-30

**Authors:** Lawrence Kwon, Jennifer Griffiths, Shlomo Greenberg, William Assante

**Affiliations:** a Department of Nephrology, Westchester Medical Center, Poughkeepsie, NY.

**Keywords:** Gordon syndrome, mineralocorticoid receptor, mineralocorticoid-dependent, NR3C2 protein, Pro701Leu missense mutation, pseudohypoaldosteronism

## Abstract

**Rationale:**

The NR3C2 gene, encoding the mineralocorticoid receptor (MR), harbors over 380 rare coding variants that significantly influence mineralocorticoid homeostasis and disease susceptibility. These variants include both loss-of-function mutations linked to renal pseudohypoaldosteronism type 1 and gain-of-function mutations associated with inherited mineralocorticoid hypertension. This case report describes the clinical features of a previously undocumented NR3C2 mutation, Pro701Leu, in a 60-year-old man presenting with hypertension and hyperkalemia.

**Patient concerns:**

A Caucasian male in his early 60 seconds presented with hypertension, hyperkalemia, and a history of chronic obstructive pulmonary disease, treated hepatitis C, bipolar disorder, and long-standing polysubstance use disorder. Despite a family history suggesting Gordon syndrome, genetic testing revealed a unique Pro701Leu mutation in the NR3C2 gene.

**Diagnoses:**

The patient was diagnosed with hypertension, hyperkalemia, and a Pro701Leu mutation in the NR3C2 gene. The mutation was identified via next-generation sequencing, revealing a missense mutation with leucine substituted for proline at codon 701 in exon 5.

**Interventions:**

Initial management included potassium level monitoring and treatment with sodium zirconium cyclosilicate. Despite these measures, hyperkalemia persisted. Furosemide and chlorthalidone were trialed, with chlorthalidone discontinued due to hyponatremia.

**Outcomes:**

The patient’s blood pressure was eventually controlled, and potassium levels normalized with continued furosemide treatment. The Pro701Leu mutation represents the first documented case linked to hyperkalemia and hypertension, suggesting an activating mutation in the MR gene.

**Lessons:**

This case highlights the importance of genetic testing in diagnosing complex cases of hypertension and hyperkalemia. The discovery of the Pro701Leu mutation in the NR3C2 gene underscores the need for further research into its pathophysiological mechanisms and therapeutic implications. Early recognition and tailored treatment can improve patient outcomes in similar cases.

## 1. Introduction

The NR3C2 gene, which encodes the mineralocorticoid receptor (MR), harbors over 380 rare coding variants that significantly influence mineralocorticoid homeostasis and disease susceptibility.^[1]^ These variants include loss-of-function mutations linked to renal pseudohypoaldosteronism type 1 and gain-of-function mutations associated with inherited mineralocorticoid hypertension, which is typically characterized by low levels of renin and aldosterone. This case report describes the clinical features of a previously undocumented NR3C2 mutation, Pro701Leu, in a 60-year-old man initially suspected of having Gordon syndrome (pseudohypoaldosteronism type 2 or familial hyperkalemia and hypertension syndrome) based on his hypertension and hyperkalemia and a family history of similar features. Genetic testing, however, identified a Pro701Leu missense mutation, with leucine substituted for proline at codon 701 in exon 5. This is the first documentation of this mutation, which should prompt further investigation into its role in hypertension and the underlying mechanisms of hyperkalemia.

## 2. Case report

A Caucasian male in his early 60s, presenting with a complex medical and social history, including chronic obstructive pulmonary disease, treated hepatitis C, bipolar disorder, and a long-standing polysubstance use disorder (tobacco, ethanol, cocaine, and heroin), was admitted to the emergency department after a self-inflicted neck injury and threatening behavior. His sister has hypertension and hyperkalemia.

Over the past decade, the patient had recurrent hospitalizations for complications from long-standing substance use, chest pain associated with cocaine use, suicide attempts, fluctuating potassium levels up to 6.1 mmol/L, cellulitis at heroin injection sites, and exacerbations of chronic obstructive pulmonary disease. His potassium values ranged from 3.2 to 6.2 mmol/L, with a median of 5.1 mmol/L. His serum creatinine consistently averaged 1.3 mg/dL, indicating early stage III chronic kidney disease as classified by the CKD-EPI equation. His mean arterial pressure, which averaged 80 mm Hg earlier in the decade, has increased to 113 mm Hg in recent years.

On this encounter, the patient’s blood pressure was 155/92 mm Hg, and he had signs of aging beyond his stated age, multiple tattoos, absence of teeth, several healing superficial lacerations, and a sutured, deeper cut on the left side of his neck. His cardiovascular examination was normal without murmurs or edema. He reported that his diet at home consists predominantly of processed foods and beer. During his hospital stay, he was placed on a diet without potassium restrictions.

On admission, potassium levels were normal at ≈ 4.0 mmol/L (Table 1). All laboratory samples were collected between 8 and 9 am while the patient was seated. During the first 5 days of hospitalization, sodium zirconium cyclosilicate was used to manage potassium levels as they gradually increased to as high as 6.2 without changes in serum creatinine. Blood pressure remained poorly controlled with metoprolol tartrate. On day 4, plasma renin activity was undetectable (below 0.6 ng/mL/h), and the plasma aldosterone concentration was low (3.7 ng/dL). Urine collected that day revealed an elevated potassium:creatinine ratio of 11.4. On day 6, repeated measurements showed similar values for renin (0.6 ng/mL/h) and aldosterone (below detection).

**Table 1:** Patient’s laboratory values.

Day	K^+^	Na^+^	Cl^‐^	TCO_2_	Cr	PRA	PAC	BP
A	4	128	94	21	1.13			163/95
–								
1	–	–	–	–	–	–	–	–
Metoprolol tartrate 25 mg (twice daily)								
2	6.2	138	101	27	1.34	–	–	155/92
Metoprolol tartrate 25 mg (twice daily)								
Sodium zirconium cyclosilicate 20 g (once)								
3	5	137	98	31	1.18	–	–	149/96
Metoprolol tartrate 25 mg (twice daily)								
4	4.9	137	99	28	1.16	< 0.6	3.7	–
Metoprolol tartrate 25 mg (twice daily)								
5	5.8	138	100	30	1.1	–	–	156/105
Sodium zirconium cyclosilicate 20 g (once)								
6	6.1	137	100	28	1.1	0.6	< 3	–
Furosemide 40 mg (once)								
Amlodipine 5 mg (once)								
7	5.4	137	97	29	1.21	–	–	116/74
Chlorthalidone 25 mg (once)								
8	5.8	135	99	26	1.32	–	–	120/80
Furosemide 40 mg (once daily)								
Sodium zirconium cyclosilicate 20 g (once)								
9	5.1	137	98	27	1.17	–	–	–
Furosemide 40 mg (once daily)								
10	5.3	137	99	27	1.31	–	–	–

A = admission, K^+^ = potassium: mmol/L, Na^+^ = sodium: mmol/L, Cl^‐^ = chloride: mmol/L, TCO_2_ = total carbon dioxide: mmol/L, Cr = creatinine: mg/dL, PRA = plasma renin activity: ng/mL/h, PAC = plasma aldosterone concentration: ng/dL, BP = blood pressure: mm Hg.

Metoprolol was withheld on day 5 due to concerns that it might be contributing to hyperkalemia; however, potassium levels increased from 5.8 to 6.1 mmol/L. Other medications administered, which are typically not associated with hyperkalemia, were budesonide-formoterol 160 µg/4.5 g inhalation twice daily; risperidone 1 mg twice daily; sertraline 100 mg once daily; tiotropium 2.5 µg inhalation once daily; oxycodone 10 mg 2–4 times daily for chronic lower back pain; and trazodone 50 mg every evening.

On day 6, the loop diuretic furosemide was administered, and by the morning of day 7, blood pressure was controlled at 116/74. However, due to a differential diagnosis of Gordon syndrome, especially since the patient disclosed that his sister also has hypertension with frequent hyperkalemia, chlorthalidone was initiated. When potassium levels increased from 5.4 to 5.8 mmol/L and sodium levels decreased from 137 to 135 mmol/L, chlorthalidone was discontinued. On hospital day 8, arterial blood pH was 7.29 and pCO_2_ 62, consistent with chronic primary respiratory acidosis with secondary metabolic acidosis (non-elevated anion gap). Furosemide 40 mg once daily was restarted, and the patient was discharged on day 10 with a prescription for furosemide 40 mg once daily. A renal ultrasound revealed normal findings.

Genetic testing was performed with a next-generation sequencing–based 385 kidney gene panel (RenasightTM test by Natera, Inc., Austin, TX). Informed consent was secured prior to collecting saliva swabs. NR3C2 variant analysis revealed a heterozygous variant, MedGen UID: 260623, located on chromosome 4 (GRCh37): g.149075965G > A NM_000901.5:c.2102C > T (p.Pro701Leu). This variant, classified as of uncertain significance, leads to a missense mutation, leucine substituting for proline at codon 701 in exon 5 of the NR3C2 gene, which is associated with autosomal dominant inheritance. Computational predictions for this variant (p.Pro701Leu) show SIFT/AGVGD scores of 1P/1B and a REVEL score of 0.29. The variant’s gnomAD score is Z = 2.10 [expected: 525.1, observed: 390] with a Grantham distance of 98.

After discharge, the patient was lost to follow-up. He had several 1-time emergency department visits for various complaints: symptoms of abdominal pain and nausea and incidents of ethanol intoxication. During these visits, the average mean arterial pressures were in the 110 seconds, potassium values were normal at 4.5 mmol/L, and creatinine values remained around his baseline of 1.3 mg/dL.

## 3. Discussion

**Figure 1.**
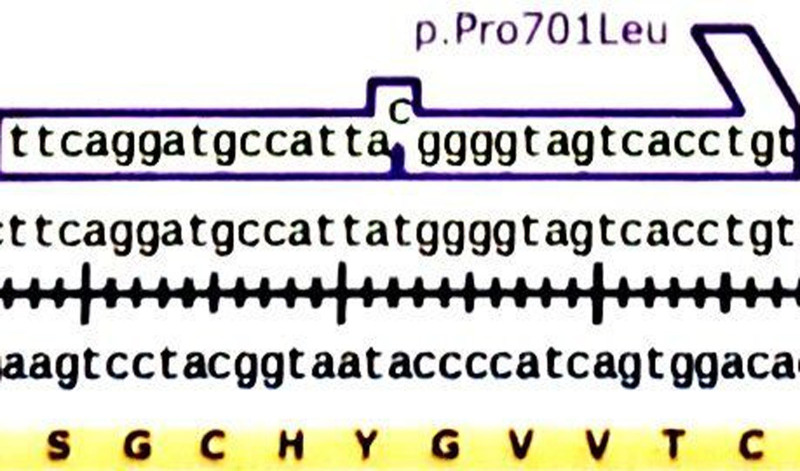
DNA sequence showing the Pro701Leu mutation in the NR3C2 gene.

This is the first published study of the Pro701Leu mutation in the NR3C2 gene, which encodes MR (Fig. 1). This mutation appears to be an activating MR mutation that results in hypertension accompanied by low levels of renin and aldosterone. Initially, we suspected Gordon syndrome, a rare inherited low-renin hypertension characterized by hyperkalemia, metabolic acidosis from type IV renal tubular acidosis, and hypoaldosteronism due to altered sodium chloride reabsorption in the distal convoluted tubule. Although mutations in Kelch-like 3 and Cullin 3 are common in Gordon syndrome,^[2]^ finding a mutation in the MR gene, NR3C2, was unexpected.

Aldosterone, produced in response to hypovolemia and hyperkalemia, is crucial for sodium reabsorption and potassium secretion in the renal distal tubule, also affecting the colon, salivary, and sweat glands. It binds to the MR in the distal nephron’s principal cells, enhancing epithelial sodium channel and Na^+^–K^+^-ATPase activity by inhibiting its degradation through specific kinase activation. MR also binds cortisol, which is converted to its inactive form by 11-beta-hydroxysteroid dehydrogenase type 2 in target tissues.^[1]^

Over 380 rare coding variants in the NR3C2 gene affect mineralocorticoid homeostasis and disease susceptibility; these variants include loss-of-function mutations that cause renal pseudohypoaldosteronism type 1 and rarer gain-of-function mutations that can lead to inherited mineralocorticoid hypertension, the best known of which is p.Ser810Leu mutation. Geller et al^[3]^ screened for the NR3C2 gene in 75 patients with early-onset severe hypertension and identified a heterozygous p.Ser810Leu mutation in a 15-year-old boy who had severe hypertension, suppressed plasma renin, and low aldosterone levels—a presentation similar to that in our patient.

The MR p.Ser810Leu mutation significantly alters the ligand-binding domain’s conformation, causing antagonist ligands such as spironolactone and progesterone to act as agonists. This mutation enables a novel van der Waals interaction between helix 5 and helix 3, stabilizing the receptor’s active conformation by replacing aldosterone’s typical anchoring at Asn770. X-ray crystallography of the mutated MR with deoxycorticosterone and progesterone shows that receptor activation can occur without traditional ligand contacts. Traditional MR antagonists are ineffective against this mutation-induced hypertension, but newer antagonists such as BR-4628 and finerenone may be effective. Moreover, carriers of the MRL810 mutation suffer severe hypertension during pregnancy, which is exacerbated by an increase in progesterone levels, causing worsening blood pressure and difficulty in renal potassium management.

Rafestin-Oblin et al^[4]^ discovered that cortisone and 11-dehydrocorticosterone, usually inactive on wild-type MR, can activate the MR L810 variant, though these levels were not measured in our patient. Kamide et al^[5]^ identified another activating mutation, F826Y, in their study of the MR gene; among the 3 affected individuals—2 females and 1 male—all exhibited normal renal function and electrolyte levels, including potassium, with renin and aldosterone levels within normal ranges in one tested individual.

It is perplexing that the activating MR mutation P701L, unlike S810L, which typically leads to hypokalemia from renal potassium wasting, would cause hyperkalemia in our patient. One possibility is that this mutation preferentially stimulates the sodium chloride cotransporter over the epithelial sodium channel, mimicking Gordon syndrome, since aldosterone in the rodent activated this cotransporter.^[6]^ However, the observation that a single day of chlorthalidone treatment in our patient raised potassium levels from 5.4 to 5.8 mmol/L challenges this hypothesis.

Over the past decade, our patient frequently presented with normal potassium levels upon arrival in the emergency department, with increases typically observed a few days into the hospital stay. Rado et al^[7]^ described a similar phenomenon as “outpatient hyperkalemia.” However, our patient experienced “inpatient hyperkalemia,” where normal potassium levels upon admission rose with a hospital diet. This case report documents the first known instance of the Pro701Leu missense mutation in the NR3C2 gene, emphasizing its significant role in the pathophysiology of hypertension and hyperkalemia. The unique presentation in our patient invites further investigation into the mutation’s mechanisms and its implications for mineralocorticoid-related disorders, warranting additional research to develop effective therapeutic strategies.

## Author contributions

**Conceptualization:** Lawrence Kwon.

**Data curation:** Lawrence Kwon, Jennifer Griffiths.

**Formal analysis:** Lawrence Kwon, Jennifer Griffiths.

**Investigation:** Lawrence Kwon, Jennifer Griffiths.

**Methodology:** Lawrence Kwon, Jennifer Griffiths.

**Visualization:** Shlomo Greenberg.

**Writing – original draft:** Lawrence Kwon.

**Writing – review & editing:** Lawrence Kwon, Jennifer Griffiths, Shlomo Greenberg, William Assante.
